# Plastidic phosphoglucomutase and ADP-glucose pyrophosphorylase mutants impair starch synthesis in rice pollen grains and cause male sterility

**DOI:** 10.1093/jxb/erw324

**Published:** 2016-09-01

**Authors:** Sang-Kyu Lee, Joon-Seob Eom, Seon-Kap Hwang, Dongjin Shin, Gynheung An, Thomas W. Okita, Jong-Seong Jeon

**Affiliations:** ^1^Graduate School of Biotechnology & Crop Biotech Institute, Kyung Hee University, Yongin 17104, Korea; ^2^Institute of Biological Chemistry, Washington State University, Pullman, WA 99164-6340, USA; ^3^Department of Southern Area Crop Science, National Institute of Crop Science, Rural Development Administration, Milyang 50424, Korea

**Keywords:** ADP-glucose pyrophosphorylase, male sterility, *Oryza sativa*, phosphoglucomutase, plastid, pollen, starch.

## Abstract

To elucidate the starch synthesis pathway and the role of this reserve in rice pollen, we characterized mutations in the plastidic phosphoglucomutase, *OspPGM,* and the plastidic large subunit of ADP-glucose (ADP-Glc) pyrophosphorylase, *OsAGPL4*. Both genes were up-regulated in maturing pollen, a stage when starch begins to accumulate. Progeny analysis of self-pollinated heterozygous lines carrying the *OspPGM* mutant alleles, *osppgm-1* and *osppgm-2*, or the *OsAGPL4* mutant allele, *osagpl4-1,* as well as reciprocal crosses between the wild type (WT) and heterozygotes revealed that loss of *OspPGM* or *OsAGPL4* caused male sterility, with the former condition rescued by the introduction of the WT *OspPGM* gene. While iodine staining and transmission electron microscopy analyses of pollen grains from homozygous *osppgm-1* lines produced by anther culture confirmed the starch null phenotype, pollen from homozygous *osagpl4* mutant lines, *osagpl4-2* and *osagpl4-3,* generated by the CRISPR/Cas system, accumulated small amounts of starch which were sufficient to produce viable seed. Such *osagpl4* mutant pollen, however, was unable to compete against WT pollen successfully, validating the important role of this reserve in fertilization. Our results demonstrate that starch is mainly polymerized from ADP-Glc synthesized from plastidic hexose phosphates in rice pollen and that starch is an essential requirement for successful fertilization in rice.

## Highlight

Analysis of rice plastidic phosphoglucomutase and ADP-glucose pyrophosphorylase mutants reveals that starch synthesis in pollen grains requires the production of glucose-1-P and ADP-glucose in the plastids.

## Introduction

Starch is one of the main storage reserves in various tissues and organs of higher plant species. The starch synthesis pathways in photosynthetic source leaves and in heterotrophic sink seed endosperms have been defined based on the analysis of a large number of mutants identified in many plant species. Such studies have revealed that the key regulatory step of the starch synthesis pathway is mediated by ADP-glucose (ADP-Glc) pyrophosphorylase (AGP) (Müller-Röber *et al.*, 1990; Okita, 1992; Tetlow *et al.*, 2004; Lee *et al.*, 2007). AGP catalyses the formation of ADP-Glc and inorganic pyrophosphate (PPi) from glucose-1-phosphate (Glc-1-P) and ATP. The resulting ADP-Glc molecule serves as the glucosyl donor for starch synthesis.

AGP functions as a heterotetrameric enzyme and is composed of two large subunits (LSs) and two small subunits (SSs) with slightly different molecular masses (Okita *et al.*, 1990; Smith-White and Preiss, 1992; Villand *et al.*, 1993; Seferoglu *et al.*, 2014). Higher plants contain multiple AGP LS and SS isoforms that are expressed in different tissues and organs and/or have different subcellular locations (Beckles *et al.*, 2001*a*, *b*; James *et al.*, 2003; Vigeolas *et al.*, 2004; Lee *et al.*, 2007; Huang *et al.*, 2014; Okamura *et al.*, 2014). In photosynthetic tissue, AGP is located in the chloroplast where it is responsible for starch synthesis, as mutations in the major leaf isoform exhibit reduced plastidic AGP activity and impaired starch synthesis (Tsai and Nelson, 1966; Dickinson and Preiss, 1969; Lin *et al.*, 1988*a*, *b*; Smith *et al.*, 1989; Wang *et al.*, 1997, 1998; Lee *et al.*, 2007; Cook *et al.*, 2012; Huang *et al.*, 2014; Okamura *et al.*, 2014). By contrast, in cereal endosperms, although there is an AGP detected in the specialized starch-containing amyloplasts, the major AGP activity is located in the cytosol (Greene and Hannah, 1998; Sikka *et al.*, 2001; James *et al.*, 2003; Johnson *et al.*, 2003; Lee *et al.*, 2007; Tuncel *et al.*, 2014).

During photosynthesis in leaves, triose-phosphates from the Calvin–Benson cycle are exported to the cytosol for the production of sucrose. A portion of the triose-phosphates are retained in the chloroplast where they are metabolized into transitory starch by co-ordinated sequential enzyme reactions involving the plastidic phosphoglucomutase (pPGM), that interconverts glucose-6-phosphate (Glc-6-P) and Glc-1-P, and plastidic AGP. Mutants with reduced pPGM activity have decreased levels of leaf starch (Caspar *et al.*, 1985; Hanson and McHale, 1988; Harrison *et al.*, 1998, 2000; Tauberger *et al.*, 2000; Fernie *et al.*, 2001). These results clearly demonstrate that Glc-1-P and, in turn, ADP-Glc is synthesized in the leaf chloroplasts. Similarly, studies in the sink organs, including seeds, of non-cereal plants also provide evidence that AGP activity is present exclusively in the plastids (Smith *et al.*, 1995; Beckles *et al.*, 2001*b*).

By contrast, a number of studies have revealed that the cytosolic AGP activity is a prerequisite for normal starch synthesis in the seed endosperm of cereal plants such as rice, barley, and maize (Greene and Hannah, 1998; Sikka *et al.*, 2001; James *et al.*, 2003; Johnson *et al.*, 2003; Lee *et al.*, 2007; Tuncel *et al.*, 2014). For instance, the low-starch endosperm mutant of barley, *Risø16*, lacks the cytosolic AGP SS isoform (Johnson *et al.*, 2003). The low-starch maize mutant *bt2* possesses a mutation in a cytosolic AGP SS (Giroux and Hannah, 1994; Hannah *et al.*, 2001). Similarly, mutants of the rice *osagps2* and *osagpl2* lack the cytosolic AGP SS and LS, respectively, and exhibit impaired starch synthesis during seed development (Lee *et al.*, 2007). In addition to null mutations, missense mutations in the *OsAGPL2* gene resulted in impaired AGP enzyme activity and, in turn, severely shrivelled, starch-deficient rice seeds (Tuncel *et al.*, 2014). These results clearly demonstrate that the cytosolic form of AGP is essential for normal starch synthesis in the developing seed endosperm of cereal plants.

In many plants including most of the world’s major cereal crops, rice, maize, wheat, and barley, starch is the main storage reserve in mature pollen grains and used for supplying energy and a carbon skeleton to support pollen germination and pollen tube growth for proper fertilization. Thus, insufficient starch synthesis in pollen grains is believed to be responsible for male sterility (Dickinson, 1968; Wen and Chase, 1999; Datta *et al.*, 2002; Wu *et al.*, 2016). As such, manipulation of the starch content in pollen grains has provided a novel strategy of inducing male sterility (Wu *et al.*, 2016). Male sterile lines are used in hybrid seed production which is widely employed for the production of high yield (Virmani and Kumar, 2009; Jeon *et al.*, 2011; Wu *et al.*, 2016). Despite its importance, little is known about the starch synthesis pathway in cereal pollen grains, an important heterotrophic organ essential for plant reproduction.

In the present study, we show that mutations in the rice plastidic PGM, *OspPGM*, and the plastidic AGP LS, *OsAGPL4*, result in starch deficiency in pollen grains and induce male sterility. These results clearly demonstrate that the main pathway of starch synthesis in this sink organ requires the synthesis of Glc-1-P and ADP-Glc in the plastid.

## Materials and methods

### Plant materials

Greenhouse-grown japonica rice [cultivar (cv.) Dongjin] WT and mutants were mainly used in the experiments. Rice plants were grown in a greenhouse at 30 °C during the day and at 20 °C at night in a light/dark cycle of 14/10h and approximately 80% humidity. Anthers from WT rice plants for RT-PCR were harvested at different development stages.

### RT-PCR analysis

Total RNA was extracted using the Trizol reagent and reverse transcription was performed with the iScript cDNA Synthesis Kit (Bio-Rad, Hercules, CA, USA). First strand cDNAs were used in RT-PCR reactions with gene-specific primers and control primers for the rice housekeeping gene *Ubiquitin5* (*OsUBQ5*; *LOC_Os01g22490*). The primers were designed in the region encompassing at least one intron for each gene to exclude genomic DNA contamination. The gene-specific primers used for RT-PCR are in Supplementary Table S1 at *JXB* online.

### Subcellular localization of the OspPGM-GFP fusion protein

The *OspPGM* full-length cDNA without stop codon was fused to the *Green Fluorescent Protein* (*GFP*) gene in-frame. The *OspPGM* full-length cDNA was amplified by PCR using the forward primer containing the *Sal*I restriction site (5′-CACCGTCGACATGGCCTCGCACGCGCTCCGCCTC-3′) and the reverse primer containing the *Sma*I restriction site (5′-TCCCCCGGGATGTTATGACAGTAGGCTTATCTC-3′). The amplified fragment was digested with the respective enzymes and cloned between the *CaMV35S* promoter and GFP of pJJ461 which was derived from pC1300intC (Ouwerkerk *et al.*, 2001). The resulting GFP fusion construct was then transformed into maize protoplasts. GFP signals were detected by excitation with the 488nm line of the argon laser and a capturing emission at 522nm by laser-scanning confocal microscopy (LSM 510 META, Carl Zeiss, Jena, Germany).

### Isolation of the *osppgm* and *osagpl4* T-DNA mutants

The *osppgm-1*, *osppgm-2*, and *osagpl4-1* mutant alleles were identified from the rice T-DNA insertion sequence database (Jeon *et al.*, 2000; Jeong *et al.*, 2006; http://www.postech.ac.kr/life/pfg/risd/index.html). Genomic DNA was isolated from young leaves of rice plants using a simple miniprep method (Chen and Ronald, 1999). Pollen grains were collected with fine pipette tips under a microscope and ground using a micropestle in a PCR tube for use as the genomic DNA template. Genotypes for the T-DNA insertion were determined by genomic DNA PCR analysis using PF1/PR1 and L1/PR1 primer sets for *osppgm-1,* PF2/PR2 and PF2/G1 sets for *osppgm-2*, and LF1/LR1 and LR1/G1 sets for *osagpl4-1*. The sequences of primers used for genotyping are listed in Supplementary Table S1.

### Reciprocal crosses

For the reciprocal crosses, *OspPGM/osppgm-1, OspPGM/osppgm-2*, and *OsAGPL4/osagl4-1* were crossed with a japonica cv. Ilpum WT rice plants. Genotypes of the crossed lines were determined by PCR using the same primer sets used for T-DNA insertions.

### Anther culture of *osppgm* mutants

To generate homozygous mutant plants of *OspPGM*, anther culture was performed as described by Eom *et al.* (2016), with slight modifications, using anthers isolated from the heterozygous plants of *OspPGM/osppgm-1* and *OspPGM/osppgm-2*. After surface sterilization of the panicles with 70% (v/v) ethanol, anthers removed from the spikelet were cultured on the N_6_ callus induction medium containing 2.0mg l^–1^ NAA, 0.2mg l^–1^ kinetin, and 5% (w/v) gelrite at 25 °C for 1 month. The induced callus was moved to the N_6_ regeneration medium containing 0.2mg l^–1^ IAA, 2.0mg l^–1^ kinetin, 2g l^–1^ casein hydrolysate, 2mg l^–1^ ABA, 40g l^–1^ maltose, and 5% (w/v) gelrite for regeneration.

### Creation of *OsAGPL4* mutants using the CRISPR/Cas system

To find an effective protospacer adjacent motif (PAM) and avoid any off-target, we screened possible target sequences using the CRISPRdirect program (Naito *et al.*, 2015; http://crispr.dbcls.jp/). Designed guide RNA (5′-GCAGTTCCTGTGGCTATTTG-3′) was cloned into an entry vector, pOs-sgRNA and then cloned into a destination vector, pH-Ubi-cas9-7, using the Gateway^TM^ system (Miao *et al.*, 2013). The resulting vector was transformed into the japonica rice cv. Dongjin by *Agrobacterium* mediation (Jeon *et al.*, 2000). The target PAM site sequence of transgenic plants was determined.

### Analysis of PGM activity by a native polyacrylamide gel electrophoresis

The in-gel PGM activity assay of the homozygous *osppm-1* mutant plants was performed as described by Egli *et al.* (2010) with slight modifications. Leaf samples were homogenized in ice-cold extraction buffer containing 100mM TRIS–HCl (pH 7.0), 10mM MgCl_2_,100mM KCl, 42mM β-mercaptoethanol, and 15% (v/v) glycerol. Proteins were resolved in non-denaturing polyacrylamide gel containing 8% (w/v) acrylamide [30% (w/v) acrylamide/0.8% (w/v) *bis*-acrylamide] and 375mM TRIS–HCl (pH 8.8). The gels were washed in the washing solution containing 50mM TRIS–HCl, pH 7.0, and 5mM MgCl_2_ for 1min and were then incubated in the staining solution containing 50mM TRIS–HCl, pH 7.0, 5mM MgCl_2_, 5.3mM Glc-1-P, 0.25mM NADP, 0.25mM NAD, 0.1mM phenazine methosulphate, 0.25mM nitroblue tetrazolium, and 40 units Glc-6-P dehydrogenase at 37 °C.

### Determination of sucrose and starch

Approximately 100mg of rice leaves and 3mg of mature anthers containing pollen grains, respectively, were harvested at the end of the day from 12-week old plants and mature flowers. The soluble sugar sucrose and insoluble starch were measured using NAD(P)H-coupled enzymatic tests in the ethanol-soluble and -insoluble fractions (Lee *et al.*, 2008). The measured metabolite contents were normalized to the leaf fresh weights.

### Pollen staining and light microscopy

For staining pollen nuclei, pollen grains were incubated in phosphate-buffered saline containing 4ng ml^–1^ Hoechst 33342 (Sigma, St Louis, MO, USA) for 1h at 65 °C. The pollen starch was stained with 10% (v/v) Lugol solution (Sigma). The nuclear- and starch-stained pollen grains were monitored under UV light and white light, respectively, with an Olympus BX61 microscope.

### Transmission electron microscopy

Pollen grains at the mature stage of development were fixed in sodium phosphate buffer (pH 7.2) containing 3% (v/v) glutaraldehyde, and then post-fixed using 2% (v/v) osmium tetroxide. After dehydration, the specimens were put into Spurr’s low-viscosity embedding mixture. Ultrathin sections (40–60nm in thickness) were stained with 2.5% (w/v) uranyl acetate and 2.5% (w/v) lead citrate aqueous solutionsbefore being examined with a transmission electron microscope, Tecnai G2 Spirit (FEI Co., Hillsboro, OR, USA).

### Measurement of AGP activity

Anthers containing pollen grains (approximately 3mg) were resuspended in 100 μl of lysis buffer containing 50mM TRIS–HCl (pH 7.6), 150mM NaCl, 5% (v/v) glycerol, 5mM EDTA, 5mM DTT, 1×proteinase inhibitors, and 1mM PMSF. The samples were collected at the bottom of a tube by centrifugation at 1 000 *g* for 5min at 4 °C. They were then ground on ice using a pestle in a 1.5ml tube and sonicated twice for 5s using a Sonic Dismembrator (Model 100, Fisher Scientific, Pittsburgh, PA, USA). Soluble proteins were obtained by centrifugation at 15 000 *g* for 10min at 4 °C and used for the enzyme assay. AGP activities were measured in the starch synthesis (ADP-Glc formation) direction as described in Hwang *et al.* (2007) with slight modifications. Briefly, the reactions were performed at 30 °C for 40min in 0.1ml of 100mM HEPES–NaOH (pH 8.0), 5mM DTT, 10mM MgCl_2_, 2mM ATP, 5mM 3-phosphoglycerate, 0.15 units/reaction inorganic pyrophosphatase (Sigma), 0.4mg ml^–1^ BSA, 2mM [^14^C] Glc-1-P (517 dpm nmol^–1^). [^14^C] ADP-Glc formation was measured using the Tri-Carb 2100TR Liquid Scintillation Counter (PerkinElmer, Boston, MA, USA).

## Results

### Identification of a rice plastidic phosphoglucomutase gene highly expressed at the starch synthesis stage during pollen development

**Fig. 1.**
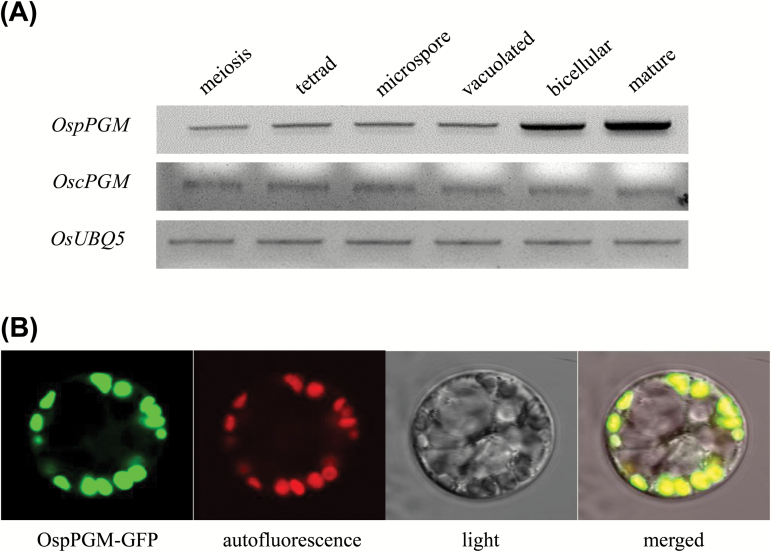
Expression profile of two rice phosphoglucomutase genes (A) and subcellular localization of OspPGM-GFP protein (B). (A) RT-PCR analysis of *OspPGM* (*LOC_Os03g50480*) and *OscPGM* (*LOC_Os10g11140*) at different stages of pollen development. *OsUBQ5* was used as the PCR control. (B) Chloroplast localization of OspPGM–GFP fusion protein in maize protoplasts. A full-length *OspPGM* cDNA was fused in-frame to GFP and expressed under the control of the *CaMV35S* promoter. The fluorescent GFP signal, chloroplast autofluorescence, light microscope view, and a merged image are shown from left to right.

To resolve the starch synthesis pathway in rice pollen grains, it was necessary to see whether a rice PGM gene is highly expressed in the bicellular and mature stages of anthers when starch begins to accumulate (Raghavan, 1988; Yamagata *et al.*, 2010). Analysis of the rice genomic sequence identified two PGM genes, *LOC_Os03g50480* and *LOC_Os10g11140* that are predicted to encode a cytosolic isoform, OscPGM, and a plastidic isoform, OspPGM, respectively (see below). Their expression was examined by semi-quantitative RT-PCR at various stages of the developing anthers. *OspPGM* expression had increased at the bicellular pollen stage and reached a peak at the mature pollen stage (Fig. 1A). By contrast, the *OscPGM* gene was expressed weakly throughout all the stages of pollen development. This result indicates that *OspPGM* expression coincides with the onset of starch accumulation during pollen development, supporting a direct role of this enzyme in starch synthesis.

Subcellular localization prediction using the ChloroP program (http://www.cbs.dtu.dk/services/ChloroP/; Emanuelsson *et al.*, 2000) identified a putative N-terminal chloroplast transit peptide of the first 44 amino acids of OspPGM. To verify its subcellular localization, a full-length *OspPGM* cDNA was fused in-frame to the *GFP* gene under the control of the *CaMV35S* promoter of a plant expression vector. The resulting construct was expressed in maize protoplasts. Analysis of the microscope images clearly showed that GFP signals and chlorophyll autofluorescence completely overlapped (Fig. 1B). This result confirms the plastidic localization of OspPGM.

### Isolation and characterization of *OspPGM* mutants

**Fig. 2.**
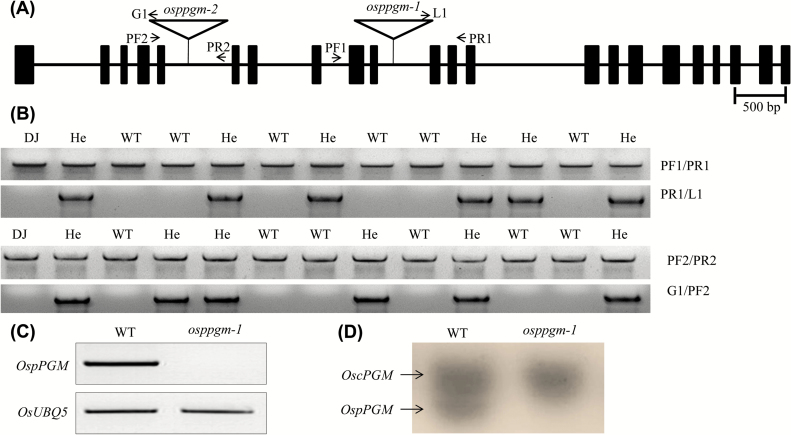
Molecular characterization of *OspPGM* mutants. (A) Schematic depiction of positions of the inserted T-DNAs in the *osppgm-1* and *osppgm-2* mutant alleles. The 22 exons of *OspPGM* are indicated by the filled boxes. Primers for genotyping are marked with arrows. In the *osppgm-1* and *osppgm-2* mutants, T-DNAs are inserted into the tenth and the fifth introns of *OspPGM,* respectively. (B) Genomic DNA PCR analysis of progeny plants of *OspPGM/osppgm-1* and *OspPGM/osppgm-2*. PF1/PR1 and L1/PR1 primers, and PF2/PR2 and G1/PF2 primers, respectively, were used for the genotyping of *OspPGM/osppgm-1* (top) and *OspPGM/osppgm-2* (bottom). DJ, mutant background genotype; WT, wild type; He, heterozygote. (C) RT-PCR analysis of the *osppgm-1* homozygote generated by anther culture. (D) In-gel activity assay of PGM following native polyacrylamide gel electrophoresis of leaf protein extracts. Two distinct activities are visible and OspPGM activity is completely missing in the *osppgm-1* homozygote as the lower faster moving band is assigned as OspPGM than the upper slower moving OscPGM.

To understand its *in vivo* role, we isolated two *OspPGM* mutant alleles, *osppgm-1* and *osppgm-2*, from our T-DNA mutant population (Jeon *et al.*, 2000; An *et al.*, 2005a, b; Jeong *et al.*, 2006). The isolated *osppgm-1* and *osppm-2* mutant alleles harbour their T-DNA insertion in the tenth and fifth introns of the *OspPGM* gene, respectively (Fig. 2A). To isolate homozygous lines for these two mutant alleles from their segregating progeny, genomic DNA PCR analysis was performed using gene- and T-DNA specific primers (Supplementary Table S1). No homozygous T-DNA insertion mutant line was found as all appeared to be WT or heterozygote (Fig. 2B). Segregation ratios of progeny of self-pollinated heterozygous plants *OspPGM/osppgm-1* and *OspPGM/osppgm-2* were found to be nearly 1:1:0 for WT, heterozygous, and homozygous genotype (37:39:0 plants from *OspPGM/osppgm-1* and 46:49:0 plants from *OspPGM/osppgm-2*) (Fig. 2B; Table 1). This raises the possibility that *osppgm-1* and *osppgm-2* mutant pollen is sterile due to the loss of Glc-1-P synthesis in the plastid.

**Table 1.** Distorted segregation in the progeny of self-pollinated *OspPGM/osppgm-1*, *OspPGM/osppgm-2*, and *OsAGPL4/osagpl4-1* plants Genotypes of progeny plants were determined by PCR using gene- and T-DNA-specific primers.

Parent plant	Observed/expected genotype of progeny in % (Observed/analysed plants in number)		
*OspPGM/osppgm-1*	*OspPGM/OspPGM* 48.7/25 (37/76)	*OspPGM/osppgm-1* 51.3/50 (39/76)	*osppgm-1/osppgm-1* 0/25 (0/76)
*OspPGM/osppgm-2*	*OspPGM/OspPGM* 48.4/25 (46/95)	*OspPGM/osppgm-2* 51.6/50 (49/95)	*osppgm-2/osppgm-2* 0/25 (0/95)
*OsAGPL4/osagpl4-1*	*OsAGPL4/OsAGPL4* 48.5/25 (50/103)	*OsAGPL4/osagpl4-1* 51.5/50 (53/103)	*osagpl4-1/osagpl4-1* 0/25 (0/103)

In order to determine whether the gametophytic defect is caused by a male or a female organ, we performed reciprocal crosses between the WT and each of the heterozygotes *OspPGM/osppgm-1* or *OspPGM/osppgm-2*, respectively. When *OspPGM/osppgm-1* or *OspPGM/osppgm-2* was used as the pollen donor, none of the F_1_ plants yielded the heterozygous *OspPGM/osppgm-1* or *OspPGM/osppgm-2* genotype. However, when the heterozygous lines were used as the female part, the genotypes of F_1_ plants were segregated nearly to 1:1 for WT and heterozygote for both mutant alleles (Table 2), clearly indicating that impaired pPGM activity in rice pollen caused male sterility.

**Table 2.** Results of reciprocal crosses between each of *OspPGM/osppgm-1, OspPGM/osppgm-2* or *OsAGPL4/osagl4-1* and wild-type plants Genotypes of F_1_ plants were determined by PCR using gene- and T-DNA-specific primers.

Genetic cross		Observed/expected genotype of progeny in % (Observed/analysed plants in number)	
Paternal parent	Maternal parent		
*OspPGM/osppgm-1*	*OspPGM/OspPGM*	*OspPGM/OspPGM* 100/50 (93/93)	*OspPGM/osppgm-1* 0/50 (0/93)
*OspPGM/OspPGM*	*OspPGM/osppgm-1*	*OspPGM/OspPGM* 51.5/50 (52/101)	*OspPGM/osppgm-1* 48.5/50 (49/101)
*OspPGM/osppgm-2*	*OspPGM/OspPGM*	*OspPGM/OspPGM* 100/50 (97/97)	*OspPGM/osppgm-2* 0/50 (0/97)
*OspPGM/OspPGM*	*OspPGM/osppgm-2*	*OspPGM/OspPGM* 48.1/50 (52/108)	*OspPGM/osppgm-2* 51.9/50/(56/108)
*OsAGPL4/osagl4-1*	*OsAGPL4/OsAGPL4*	*OsAGPL4/OsAGPL4* 100/50 (87/87)	*OsAGPL4/osagl4-1* 0/50 (0/87)
*OsAGPL4/OsAGPL4*	*OsAGPL4/osagl4-1*	*OsAGPL4/OsAGPL4* 48.4/50 (45/93)	*OsAGPL4/osagl4-1* 51.4/50 (48/93)

### Genetic complementation of the *OspPGM* mutant

To determine if the *osppgm* mutation is responsible for the male sterile phenotype, we introduced the WT *OspPGM* cDNA under the control of the constitutive maize (*Zea mays*) *Ubiquitin1* (*ZmUbi1*) promoter into hygromycin-resistant scutellum-derived calli from the *OspPGM/osppgm-1* heterozygous seeds by *Agrobacterium*-mediated transfer. We obtained three *Ubi*:*OspPGM* transgenic lines by the *Phosphomannose Isomerase*/mannose selection system (Eom *et al.*, 2011). Genomic DNA PCR analysis indicated that these lines were all heterozygous for the *osppgm-1* allele and harboured the WT *OspPGM* cDNA transgene. We then analysed progeny plants of the transgenic lines and found a number of *OspPGM* cDNA-carrying *osppgm-1/osppgm-1* homozygous plants among the progeny of the independent transgenic lines (see Supplementary Fig. S1A at *JXB* online). Expression of the *OspPGM* transgene was confirmed in the subsequently selected complemented plants (i.e. *osppgm-1/osppgm-1*//*Ubi*:*OspPGM/Ubi*:*OspPGM*; hereafter referred to as Comp) (Supplementary Fig. S1B). This result demonstrates that the defect in fertility of *osppgm-1* mutant pollen was rescued by overexpression of the WT *OspPGM* in the complemented lines. The line (Comp #4) with the highest expression of the introduced *OspPGM* transgene was chosen for further analysis (Supplementary Fig. S1B).

### Production and phenotype of homozygous *osppgm* mutant

To examine in detail the role of OspPGM in developing pollen, as well as in other organs including the photosynthetic leaf, we generated homozygous *osppgm* mutants by anther culture. In total, 29 and 17 independent plants were produced from the anthers of *OspPGM/osppgm-1* and *OspPGM/osppgm-2*, respectively. We successfully generated 14 normal green *osppgm-1/osppgm-1* homozygotes and 15 WT at a segregation ratio close to 1:1. By contrast, all regenerated WT and *osppgm-2/osppgm-2* homozygotes were albino, a phenotype most likely due to a background mutation. Thus, we only analysed the *osppgm-1/osppgm-1* homozygous plants.

RT-PCR analysis confirmed that the *OspPGM* endogenous mRNA expression was completely abolished in *osppgm-1/osppgm-1* plants (Fig. 2C). In order to determine the loss of its enzymatic activity, crude extracts of the leaf tissues were separated by a native polyacrylamide gel electrophoresis followed by staining for PGM activity (Egli *et al.*, 2010). In the WT extract, two distinct bands were observed but only the upper band was visible in the *osppgm-1/osppgm-1* mutant. Because the rice genome contains two PGMs, cytosolic and plastidic isoforms, the lower faster-moving band is attributable to the plastidic form OspPGM as it is missing in the *osppgm-1/osppgm-1* mutant, while the upper slower-moving band is the cytosolic OscPGM (Fig. 2D). Based on these results, we concluded that pPGM activity was abolished in the *osppgm-1/osppgm-1* plants.

**Fig. 3.**
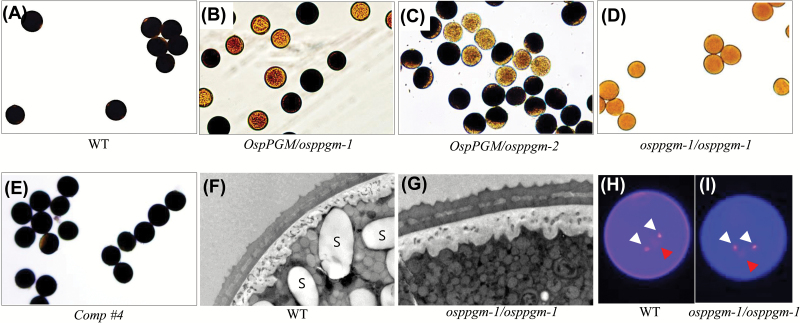
Phenotype of mature pollen grains of *osppgm-1/osppgm-1, OspPGM/osppgm-1*, *OspPGM/osppgm-2,* and *Comp #4* complemented plant. (A–E) Iodine-stained pollen. (F, G) Transmission electron microscopy images of pollen. (H, I) Nuclear stained pollen by Hoechst 33342. (A, F, H) Wild type; (B) *OspPGM/osppgm-1* (C) *OspPGM/osppgm-2* (D, G, I) *osppgm-1* homozygote, and (E) complemented line # 4. White arrowhead, generative nucleus; red arrowhead, vegetative nucleus. WT, wild type; S, starch.

To understand the male sterile phenotype of *osppgm* mutants, we then examined the starch-staining capacity of pollen grains from *osppgm-1/osppgm-1* homozygous plants as well as those from *OspPGM/osppgm-1, OspPGM/osppgm-2,* and Comp #4. We found that all the pollen grains of *osppgm-1/osppgm-1* were not stained by iodine solution, indicating that they contain little or no starch. By contrast, all pollen grains of Comp #4 and about 50% of the pollen from *OspPGM/osppgm-1* and *OspPGM/osppgm-2* stained dark with iodine (Fig. 3A–E). In addition, transmission electron microscopy analysis verified that the *osppgm-1/osppgm-1* pollen did not contain any starch granules, while the WT had large ones (Fig. 3F, G). Based on nuclear staining with Hoechst 33342, all WT and mutant pollen contained one vegetative nucleus and two generative nuclei (Fig. 3H, I) suggesting that *osppgm* mutant pollen developed normally but did not accumulate any starch. This result clearly supports the view that *osppgm* pollen grains develop normally but cannot fertilize due to limited accumulation of reserve starch.

The plastidic PGM is known to be a key enzyme for starch synthesis during the day in the mesophyll cells of a photosynthetic leaf. Thus, we determined the starch content in the leaves of the *osppgm-1/osppgm-1* homozygote, WT, and Comp #4 plants. The second leaves collected from the top of the plants immediately prior to heading were analysed at the end of the day. Starch did not accumulate in the leaf of *osppgm-1/osppgm-1* (Supplementary Fig. S2A), while WT and Comp #4 accumulated starch normally. This starch-deficient phenotype of *osppgm-1/osppgm-1* is identical to that observed in the chloroplast PGM mutant lines from other plant species (Caspar *et al.*, 1985; Hanson and McHale, 1988; Harrison *et al.*, 1998, 2000; Tauberger *et al.*, 2000; Fernie *et al.*, 2001).

As newly fixed carbon is normally partitioned between sucrose and starch, we determined the sucrose levels in these rice lines. Sucrose levels were found to be higher in the *osppgm-1/osppgm-1* than in the control lines (Supplementary Fig. S2B), while no significant difference in the levels of sucrose and starch between WT and Comp #4 was evident. Our observations are consistent with the results of other reported pPGM mutants where sucrose levels are elevated when starch synthesis is suppressed (Caspar *et al.*, 1985; Hanson and McHale, 1988; Fernie *et al.*, 2001).

Although carbon partitioning is clearly affected, we observed no detectable differences in the size, morphology or development of *osppgm-1/osppgm-1* plants compared with the controls (Supplementary Fig. S2C) under our growth conditions, with the exception that all *osppgm-1/osppgm-1* plants generated by anther culture remained sterile over the many years that they were maintained. No visible phenotypic differences in vegetative growth were observed for the starchless rice mutants, *osagps2* and *apl1* (Lee *et al.*, 2007; Rösti *et al.*, 2007). The absence of any effect on growth and development by leaf starch deficiency in rice may simply be due to the fact that this plant has limited starch synthesis capacity and that its growth relies more on the synthesis of sucrose (Lee *et al.*, 2008, 2014).

### Identification of an ADP-glucose pyrophosphorylase gene highly expressed in the starch synthesis stage of pollen

**Fig. 4.**
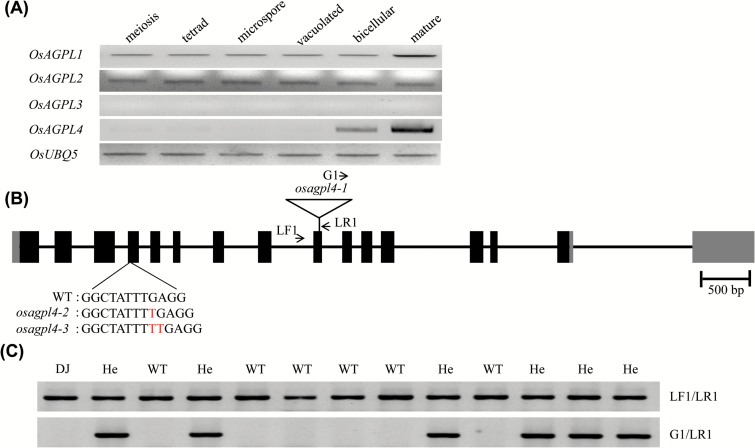
Expression of rice OsAGP large subunit genes during pollen development (A) and molecular characterization of *osagpl4* mutant (B, C). (A) RT-PCR analysis of *OsAGPL* genes. Transcript levels of different stage tissues are comparable for each gene in the PCR reactions. *OsUBQ5* was used as the PCR control. (B) Schematic depiction of positions of the T-DNA insertion in the *osagpl4-1*. The 16 exons of *OsAGPL4* are shown by filled boxes. Primers for genotyping are marked with arrows. The other two alleles, *osagpl4-2* and *osagpl4-3,* generated by the CRISPR/Cas system have one and two nucleotide insertions, respectively. (C) Genomic DNA PCR analysis of progeny of the self-pollinated *OsAGPL4/osagpl4-1*. LF1/LR1 and G1/LR1 primer sets were used for genotyping of the wild type *OsAGPL4* copy and T-DNA insertion allele, respectively. DJ, mutant background genotype; WT, wild type; He, heterozygote.

The aforementioned results indicate that the plastidic PGM-deficient rice mutants exhibit impaired starch synthesis in pollen grains that renders them male sterile. This suggests that the hexose phosphate pool within the pollen plastids is used for ADP-Glc synthesis, unlike a large cytosolic ADP-Glc pool used for starch synthesis in cereal endosperm including rice (Sullivan *et al.*, 1995; Sikka *et al.*, 2001; Lee *et al.*, 2007; Cakir *et al.*, 2016). This prompted us to identify a plastidic AGP isoform that functions mainly in starch synthesis of rice pollen. For this, we analysed the expression of the four rice AGP LS isoform genes at various stages of the developing anthers. Such analysis revealed that *OsAGPL4* (*LOC_Os07g13980*) began to be expressed at the bicellular stage and reached its highest level at the mature stage when the pollen grains accumulate starch (Fig. 4A). Consistently, expression analysis of publically available datasets from the Rice Massively Parallel Signature Sequencing (rice MPSS; https://mpss.danforthcenter.org/rice/mpss_index.php) and the NCBI Gene Expression Omnibus (GEO; http://www.ncbi.nlm.nih.gov/geo) supported the pollen preferential expression of *OsAGPL4* (Supplementary Fig. S3). The other AGP LS isoforms were constitutively expressed at much lower levels, with the exception of *OsAGPL3* whose transcript was not detectable at all of the stages examined. We had previously shown that the OsAGPL4 is located in the plastids (Lee *et al.*, 2007).

### Isolation and characterization of the *OsAGPL4* mutant

We had previously demonstrated that the rice *OsAGPL2* mutant line exhibits a shrunken seed endosperm phenotype (Lee *et al.*, 2007). The *OsAGPS2* mutant, lacking both alternative-spliced transcripts encoding the plastidic (*OsAGPS2a*) and cytosolic (*OsAGPS2b*) forms, produced starchless leaves and a shrunken endosperm phenotype (Lee *et al.*, 2007). An *OsAGPL3* mutant, *apl1*, also showed impaired starch synthesis in leaves (Rösti *et al.*, 2007) while an *OsAGPL1* mutant, *apl3*, lacked starch in the culm (Cook *et al.*, 2012). As none of these mutants shows any distortion in progeny segregation or male sterile phenotype, we isolated a mutant of *OsAGPL4*, highly expressed in the starch synthesis stage of pollen, *osagpl4-1*, from the rice T-DNA mutant library (Jeon *et al.*, 2000; An *et al.*, 2005*a*, *b*; Jeong *et al.*, 2006).

The *osagpl4-1* mutant allele harboured a T-DNA insertion in the ninth exon (Fig. 4B). PCR analysis of the genomic DNA of progeny plants of the *OsAGPL4* heterozygous mutant using gene- and T-DNA specific primers (Supplementary Table S1) revealed no *osagpl4-1/osagpl4-1* homozygous individuals. Instead, progeny segregated 1:1 (50:53 plants) for WT and heterozygote (Fig. 4C; Table 1). This raised the possibility that *osagpl4-1* mutant pollen is sterile possibly due to a defect in starch synthesis. We performed reciprocal crosses between the WT and *OsAGPL4/osagpl4-1*. When the female parent was the heterozygous line, F_1_ genotypes were segregated nearly 1:1 for WT and heterozygote (Table 2). By contrast, the other cross did not transmit the *osagpl4-1* allele to their F_1_ plants. This result strongly indicates that the loss of *OsAGPL4* in rice pollen caused male sterility.

**Fig. 5.**
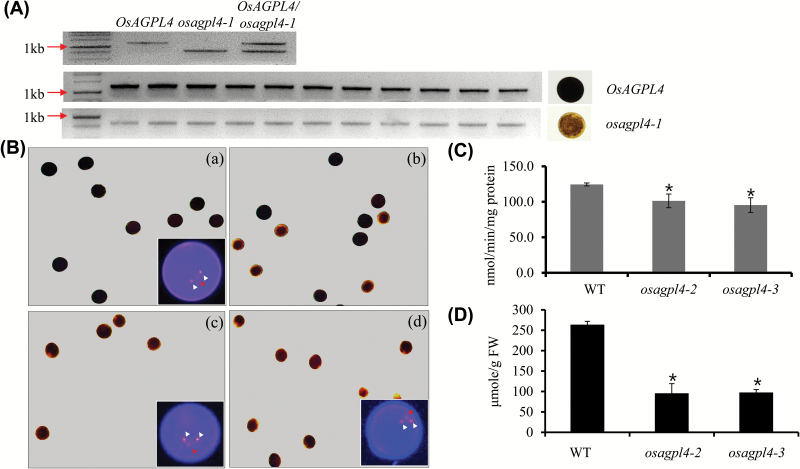
Molecular, phenotypic, and biochemical analysis of *osagpl4* mutant pollen. (A) Genomic DNA PCR analysis of individual pollen grains of *OsAGPL4/osagpl4-1.* (Top) The LF1/LR1 primer set was used for the wild-type *OsAGPL4* gene, and the G1/LR1 set for *osagpl4-1.* (Middle and bottom) LF1, LR1, and G1 primers were mixed in the PCR reactions from genomic DNAs of individual pollen of *OsAGPL4/osagpl4-1.* All pollen grains darkly stained by iodine solution contain the wild-type *OsAGPL4* (middle) and all weakly stained pollen grains carry *osagpl4-1* (bottom). (B) Iodine stained pollen. (a) Wild type, (b) *OsAGPL4/osagpl4-1,* (c) *osagpl4-2/osagpl4-2,* (d) *osagpl4-3/osagpl4-3*. Pollen grains stained with Hoechst 33342 are shown in insets for the wild type and two homozygous mutants, *osagpl4-2/osagpl4-2* and *osagpl4-3/osagpl4-3*, generated by the CRISPR/Cas system. White arrowhead, generative nucleus; red arrowhead, vegetative nucleus. (C) AGP activity of mature anthers of the wild type and homozygous *osagpl4-2* and *osagpl4-3* lines. (D) Starch contents of the mature anthers of the wild type and homozygous *osagpl4-2* and *osagpl4-3* lines. WT, wild type. Each data point represents the mean ±SD from at least three different plants. *P <0.01.

We next examined starch levels by iodine staining of the pollen grains of the *OsAGPL4/osagpl4-1* heterozygous plant, and found that about a half of the pollen was strongly stained and the other weakly stained, while all of that from the WT stained dark (Fig. 5B). To examine if the level of starch staining is correlated with the *OsAGPL4* genotype of each pollen, PCR analysis was performed with genomic DNAs isolated from individual pollen grains. The result showed that all dark-stained pollen produced the WT copy of *OsAGPL4*, while the weakly stained pollen amplified the *osagpl4-1* allele (Fig. 5A). This indicates that impaired function of *OsAGPL4* reduces starch synthesis.

### Production and characterization of *osagpl4* homozygous mutants

To understand further the role of *OsAGPL4* in the starch synthesis of rice pollen, we adopted the CRISPR/Cas system, as an alternative for anther culture, to produce homozygous mutant lines. A region of the fourth exon was selected as the target of guide RNA. Of 43 independent transgenic lines, we found two lines, *osagpl4-2* and *osagpl4-3*, with their homozygous mutant alleles at the target site. The *osagpl4-2* and *osagpl4-3* alleles had one and two nucleotide insertions, respectively (Fig. 4B). Pollen of the homozygous *osagpl4-2* and *osagpl4-3* lines was examined by starch staining. The results showed that all pollen grains from both lines stained weakly (Fig. 5B). In nuclear staining with Hoechst 33342, all pollen grains carrying the WT OsAGPL4, *osagpl4-2* or *osagpl4-3* allele contained one vegetative nucleus and two generative nuclei (Fig. 5B, insets), suggesting that the *osagpl4* mutant pollen developed normally.

The AGP activities of two homozygous *osagpl4* lines were measured according to the previously described method using ^14^C-labelled Glc-1-P (Hwang *et al.*, 2007). We collected the anthers containing mature pollen grains from the WT, *osagpl4-2*, and *osagpl4-3* lines. The levels of AGP activity in *osagpl4-2* and *osagpl4-3* were found to be significantly reduced by 18.6% and 23.4%, respectively, in the mature anthers, compared with that of the WT (Fig. 5C). Our quantitative measurement clearly confirmed that the levels of starch in *osagpl4-2* and *osagpl4-3* were significantly reduced by about 65% in the mature anthers, compared with that of the WT (Fig. 5D). This result demonstrates that OsAGPL4 is a major AGP LS isoform in rice pollen. The other AGP isoforms, which were also expressed during pollen development (Fig. 4A), are probably responsible for the remaining AGP activity in the mutants. The AGP activity from the anther walls could also contribute to the AGP activity in the mutants.

Interestingly, the homozygous alleles of *osagpl4-2* and *osagpl4-3* bore a number of seeds, resulting in about 10–20% fertility in our growth conditions. Genomic DNA PCR analysis of these progeny plants confirmed their homozygous mutant genotypes. This result suggests that *osagpl4* mutant pollen with a reduced starch content retained some fertilization ability but cannot compete with the WT pollen when both are present in the heterozygous plants.

## Discussion

Many major food crop plants, including rice, store starch in their pollen to provide the building blocks and energy reserves for pollen germination and subsequent elongation of the pollen tube. By contrast, *Arabidopsis thaliana*, a dicot model plant, utilizes lipid bodies as their storage reserve in pollen grains. Thus, the presence of starch in pollen grains is critical for normal pollen fertilization and it is commonly used as a viable pollen indicator, but our current understanding on how carbon is routed into starch in this vital plant organ has not been defined until now. In cereals, it is now well established that ADP-Glc, the sugar nucleotide utilized by starch synthase, is synthesized by two different pathways. In source leaves, the synthesis of ADP-Glc is restricted to the chloroplast and used for the production of transitory starch while, in the developing sink organ, seed endosperm, the synthesis of ADP-Glc involves cytosolic events. In our present study, based on our analyses of mutants of the rice plastidic PGM, *OspPGM*, and the plastidic AGP LS isoform, *OsAGPL4*, we demonstrate that starch synthesis in the sink organ pollen depends upon the pool of plastidic synthesized ADP-Glc.

### Loss-of-function of *OspPGM* results in a defect in starch synthesis and causes male sterility

In rice, pollen grains start accumulating starch at the bicellular stage and the starch levels gradually increase until maturity (Raghavan, 1988; Yamagata *et al.*, 2010). Many pollen mutants that lack starch are non-viable in the fertilization process (Min *et al.*, 2013; Zhang *et al.*, 2013; Khan *et al.*, 2015; Wu *et al.*, 2016). Because PGM is an important enzyme in starch synthesis, we showed that rice contains both cytosolic and plastidic isoforms for PGM and that the plastidic isoform gene, *OspPGM*, is highly expressed in the mature pollen stages (Fig. 1A). Native gel activity analysis of the homozygous *pPGM* mutant, *osppgm-1*, supports the view that the rice genome encodes a unique pPGM (Fig. 2D). Here, we found that the *osppgm* mutant pollen grains lack starch (Fig. 3) and were male sterile (Tables 1, 2). Direct evidence that male sterility is caused by starch deficiency in pollen is provided by the result that fertile pollen grains were produced when the *osppgm* mutation was complemented by the WT *OspPGM* gene (Supplementary Fig. S1). The male-sterile phenotype has not been reported from the pPGM mutants of other plants, including Arabidopsis that utilizes lipid bodies as a major reserve in pollen grains (Caspar *et al.*, 1985; Hanson and McHale, 1988; Harrison *et al.*, 1998, 2000; Tauberger *et al.*, 2000; Fernie *et al.*, 2001).

It has been well established that the accumulation and metabolism of leaf starch is important for the growth of dicot plant species, including Arabidopsis, as readily evident by mutations in pPGM, AGP, β-amylase, α-glucan water dikinase (GWD1), and the maltose transporter (Caspar *et al.*, 1985, 1991; Lin et al., 1988*a*, *b*; Niittylä *et al.*, 2004; Fulton *et al.*, 2008). Moreover, the elevation of leaf starch was demonstrated to enhance photosynthetic capacity and plant growth in rice as well as in Arabidopsis (Gibson *et al.*, 2003, 2011). Unlike Arabidopsis, however, rice leaves normally accumulate relatively little starch. Our mutant analyses of the rice cytosolic fructose 1,6 *bis*phosphatase and the plastidic triose phosphate/phosphate translocator demonstrated that rice has a limited capacity for starch synthesis in the source leaf (Lee *et al.*, 2008, 2014). Hence, the impairment of the synthesis of transitory leaf starch mediated by mutations in *apl1*, an *OsAGPL3* mutant, and *osagps2*, an *OsAGPS2* mutant, would not be expected to disrupt normal plant growth (Lee *et al.*, 2007; Rösti *et al.*, 2007). Similarly, the rice leaf starch excess1 (*lse1*), a *GWD1* mutant, grew normally unlike the mutants of the Arabidopsis homologous gene (Hirose *et al.*, 2013). In the present study, the *osppgm-1* homozygous mutant did not synthesize starch in the source leaf during the day and grew normally (Supplementary Fig. S2). This result strongly supports our previous results that rice plant growth relies more on the synthesis of sucrose under normal condition.

### *OsAGPL4* encodes a major large subunit of AGP for starch synthesis in rice pollen

OspPGM is the enzyme isoform that converts Glc-6-P to Glc-1-P in the plastids of rice. The finding that *osppgm* mutants abolish starch synthesis in rice pollen grains supports the view that ADP-Glc, the substrate for starch synthesis, is mainly synthesized from Glc-1-P in the plastids of pollen rather than in the cytosol. To verify this hypothesis, we identified the plastidic *OsAGPL4* as the dominant AGP LS which is preferentially expressed at the bicellular and mature stages of pollen development (Fig. 4A). Its localization was previously determined as plastidic (Lee *et al.*, 2007). Our observation of *OsAGPL4* alleles, *osagpl4-1*, *osaspl4-2*, and *osagpl4-3*, showed that *OsAGPL4* deficiency resulted in reduced starch content as indicated by starch staining and quantitative measurement (Fig. 5B, D) and mediated a male sterile phenotype (Tables 1, 2). AGP activity in anthers containing *osagpl4* mutant pollen was significantly reduced (Fig. 5C), although substantial amounts of enzyme were probably contributed by OsAGPL1, another plastidic AGP LS isoform (Lee *et al.*, 2007), which may compensate starch synthesis in *osagpl4* mutant pollen. Previous mutant analysis on the rice AGP genes from our and other groups did not show any male sterile phenotype (Lee *et al.*, 2007; Rösti *et al.*, 2007; Cook *et al.*, 2012; Okamura *et al.*, 2014), supporting that OsAGPL4 is critical for starch synthesis in rice pollen.

### A model for the starch synthesis pathway in rice pollen

**Fig. 6.**
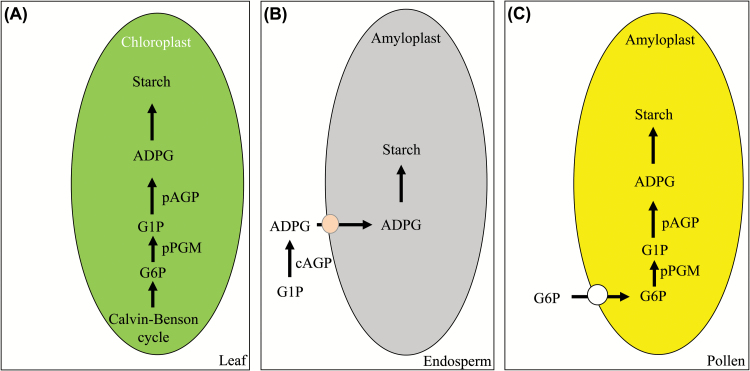
Proposed pathways of starch synthesis in the plastid of leaf (A), endosperm (B), and pollen grains (C) in rice. (A) In rice leaves, photoassimilates produced from the Calvin–Benson cycle is stored as transitory starch by the co-ordinated action of the plastidic OspPGM and OsAGP isoforms, mainly OsAGPS2a and OsAGPL3. Starch synthesis in rice leaves depends on the synthesis of ADP-Glc in the chloroplast. (B) In rice seed endosperm, the cytosolic AGP isoforms, OsAGPS2b/OsAGPL2 complex, constitute the dominant enzyme activity in starch synthesis. Starch synthesis in the seed endosperm mainly depends on the cytosolic ADP-Glc synthesis. Developing endosperms are exposed to low oxygen stress as the seeds mature. Thus, the cytosolic AGP activity enforces the UDP-glucose pyrophosphorylase metabolic flux using PPi as an alternative to the ATP-dependent enzyme/pathway. (C) In rice pollen, Glc-6-P is converted to Glc-1-P via pPGM and Glc-1-P is then converted to ADP-Glc in the amyloplasts. This suggests that starch synthesis in rice pollen grains mainly depends on the plastidic ADP-Glc synthesis, similar to that seen in rice leaves. This model suggests the potential roles of the plastidic ATP/ADP translocator and Glc-6-P/phosphate translocator in rice pollen grains. pAGP, plastidic ADP-Glc pyrophosphorylase; cAGP, cytosolic ADP-Glc pyrophosphorylase; pPGM, plastidic phosphoglucomutase; ADPG, ADP-Glc; G1P, Glc-1-P; G6P, Glc-6-P. (Online in colour.)

Previous and current results suggest that, in rice leaves, the photoassimilates produced from the Calvin–Benson cycle is stored as transitory starch by the co-ordinated function of both plastidic OspPGM and OsAGP isoforms, mainly OsAGPS2a and OsAGPL3 (Fig. 6A). Each of their mutations, *osppgm*, *osagps2*, and *apl1*, caused starch deficiency in rice leaves (Supplementary Fig. S2) (Lee *et al.*, 2007; Rösti *et al.*, 2007). In rice seed endosperm, the cytosolic AGP isoforms, the OsAGPS2b/OsAGPL2 complex, comprise the dominant enzyme activity in starch synthesis (Fig. 6B) (Lee *et al.*, 2007). A lesion of one of the two subunits, OsAGPL2 and OsAGPS2b, produced a shrunken seed endosperm due to a remarkable reduction in starch synthesis. Thus, starch synthesis in the rice leaf and seed endosperm, mainly depends on the plastidic and cytosolic ADP-Glc synthesis pathway, respectively.

The developing endosperms are subjected to low oxygen stress as the seeds mature (Rolletschek *et al.*, 2002, 2011). Hence, energy is produced mainly by glycolysis instead of by respiration. Cereal endosperm compensates for the reduction in energy production by conserving nucleoside triphosphates and PPi (Tuncel *et al.*, 2014; Cakir *et al.*, 2016). Sucrose imported from source leaves is broken down by sucrose synthase, which forms UDP-glucose (UDP-Glc) and fructose. UDP-Glc, together with PPi, are then acted upon by UDP-Glc pyrophosphorylase (UGPase) to form Glc-1-P and UTP. The newly synthesized Glc-1-P, together with ATP, is used to synthesize ADP-Glc by AGP. In this series of reactions, the high-energy PPi is consumed in the UGPase reaction but is re-synthesized in the AGP step. Although ATP is consumed UTP is synthesized, thereby conserving nucleoside triphosphates.

Our present finding proposes a model for the starch synthesis pathway in the sink organ pollen that has been unknown until now (Fig. 6C). Lesions in the plastidic isoforms, OsAGPL4 and OspPGM, impair starch synthesis that, in turn, causes sterile pollen (Figs 2–5). This result indicates that Glc-6-P is converted to Glc-1-P via pPGM and that Glc-1-P is then converted to ADP-Glc in the amyloplasts of rice pollen. This suggests that starch synthesis in rice pollen mainly depends on the plastidic ADP-Glc synthesis, as in photosynthetic leaves. In the photosynthetic leaves, ATP for starch synthesis is supplied by photophosphorylation during photosynthesis. In the heterotrophic organ pollen, ATP is supplied through respiration in the mitochondria, suggesting a potential involvement of a plastidic ATP/ADP translocator protein (AATP). In addition, it is likely that Glc-6-P is preferentially imported into the amyloplasts of rice pollen grains by a Glc-6-P/phosphate translocator (GPT). This is consistent with the results from dicot plants, including Arabidopsis, that plastids of heterotrophic tissues preferentially import Glc-6-P (Niewiadomski *et al.*, 2005; Weber *et al.*, 2005; Facchinelli and Weber, 2011). The Arabidopsis *GPT1* mutants caused severe defects especially during pollen development due to less lipid body formation (Niewiadomski *et al.*, 2005). Putative plastidic AATPs and GPTs have previously been identified in rice (Toyota *et al.*, 2006). Expression analysis with publicly available microarray datasets indicated that two AATP genes (*LOC_Os01g45910* and *LOC_Os02g11740*) and two GPT genes (*LOC_Os08g08840* and *LOC_Os07g33910*) are expressed in developing rice pollen (Supplementary Fig. S3). Therefore, it would be valuable in future to determine a role of these plastidic translocators that function in rice pollen.

### The *OsAGPL4* mutant may be used for the production of hybrid rice

Hybrid breeding exploits the increased vigour or heterosis of F_1_ hybrid plants. Cytoplasmic male sterility, photoperiod-sensitive genic male sterility, and thermo-sensitive genic male sterility systems are all used for hybrid rice breeding (Virmani and Kumar, 2009; Jeon *et al.*, 2011). An alternative strategy for engineering male sterility has recently been developed by simply abolishing starch accumulation in maize pollen resulting from the expression of α-amylase (Wu *et al.*, 2016). In the present study, the homozygous *osagl4-2* and *osagpl4-3* lines created by CRISPR/Cas yielded a considerable number of homozygous mutant seeds. We showed that the heterozygous *OsAGPL4/osagpl4-1* did not produce any *osagpl4-1/osagpl4-1* homozygous progeny plants (Table 1), and that the reciprocal cross experiment confirmed that the *osagpl4-1* allele could not be transmitted to the next generation through pollen of the heterozygote *OsAGPL4/osagpl4-1* (Table 2). The production of homozygous progeny of *osagpl4-2* and *osagpl4-3* is not surprising, considering that the mutant pollen accumulated a low level of starch. This result most likely suggests that *osagpl4* mutant pollen retains their fertilization ability, although they are unable to compete against WT pollen. This raises an alternative way for engineering male sterility by creating homozygous mutants of a pollen-specific gene that codes for a major carbon metabolic gene of starch synthesis with functionally redundant isoforms. Nevertheless, this possibility needs to be investigated in the future.

In conclusion, our present study identified the OspPGM and OsAGP isoforms that are essential for starch synthesis in rice pollen grains. We also established a model of starch synthesis among different organs during rice growth and development. The new information provided in this study may also facilitate the fine control of the starch synthesis pathway in the future. This has the potential to enhance fertility in order to increase yield productivity under environmental stress conditions and to control the male sterility trait in hybrid rice production.

## Supplementary data

Supplementary data are available at *JXB* online.

Table S1. List of PCR primers used in this study.

Figure S1. Genomic DNA PCR (A) and RT-PCR (B) analysis of genetically complemented lines of *osppgm-1* with wild type *OspPGM* copy under the control of the maize *Ubi1* promoter.

Figure S2. Metabolite and phenotypic analysis of *osppgm-1/osppgm-1* mutant rice plant generated by anther culture.

Figure S3. Digital expression profile of *OsAGPL4,* the putative plastidic ATP/ADP translocator, and Glc-6-P/phosphate translocator genes.

Supplementary Data
