# Supplementary material for: Plastidic phosphoglucomutase and ADP-glucose pyrophosphorylase mutants impair starch synthesis in rice pollen grains and cause male sterility
Source: J Exp Bot. 2016 Sep 1;67(18):5557–69. doi: 10.1093/jxb/erw324 (PMC5049399; doi:10.1093/jxb/erw324)
Supplement: Supplementary Data [file supp_67_18_5557__index.html]

Plastidic phosphoglucomutase and ADP-glucose pyrophosphorylase mutants impair starch synthesis in rice pollen grains and cause male sterility — Plastidic phosphoglucomutase and ADP-glucose pyrophosphorylase mutants impair starch synthesis in rice pollen grains and cause male sterility — Supplementary Data 

# Plastidic phosphoglucomutase and ADP-glucose pyrophosphorylase mutants impair starch synthesis in rice pollen grains and cause male sterility

## Supplementary Data

Data files

- Supplementary\_Figures\_S1\_S3\_Table\_S1.pdf - Supplementary Data
