# Supplementary material for: Plastidic phosphoglucomutase and ADP-glucose pyrophosphorylase mutants impair starch synthesis in rice pollen grains and cause male sterility
Source: J Exp Bot. 2016 Sep 1;67(18):5557–69. doi: 10.1093/jxb/erw324 (PMC5049399; doi:10.1093/jxb/erw324)
Supplement: Supplementary Data [file supp_erw324_Supplementary_Figures_S1_S3_Table_S1.pdf]

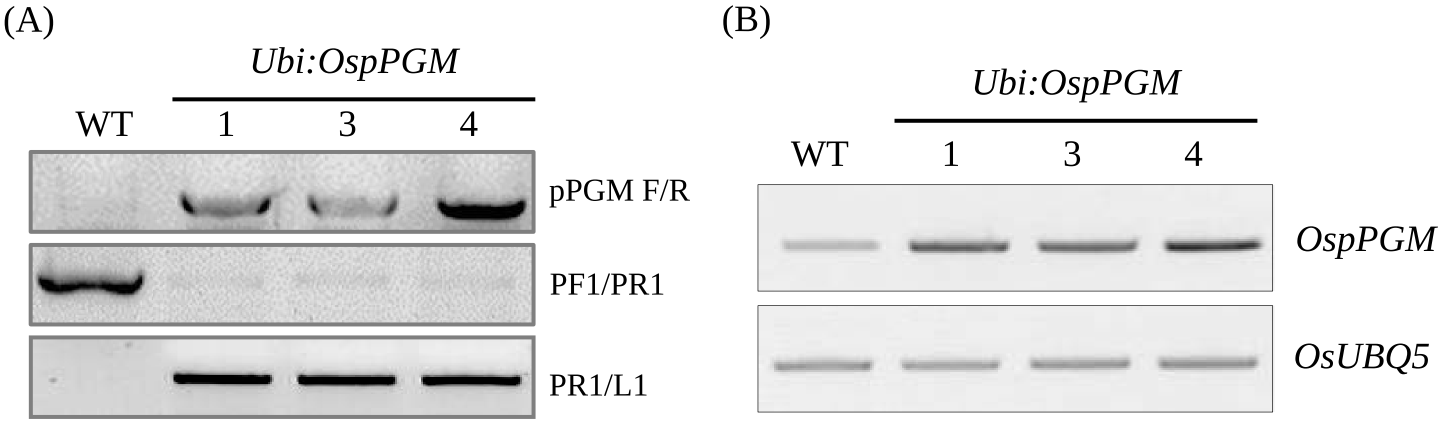

Fig. S1. Genomic DNA PCR (A) and RT-PCR (B) analysis of genetically complemented lines of *osppgm-1* with wild type *OspPGM* copy under the control of the maize *Ubi1* promoter. pPGM F/R primer set is used for *OspPGM* cDNA, PF1/PR1 set for wild type genomic copy, and PR1/L1 set for T-DNA insertion. *OsUBQ5* was used as PCR control. WT, wild type

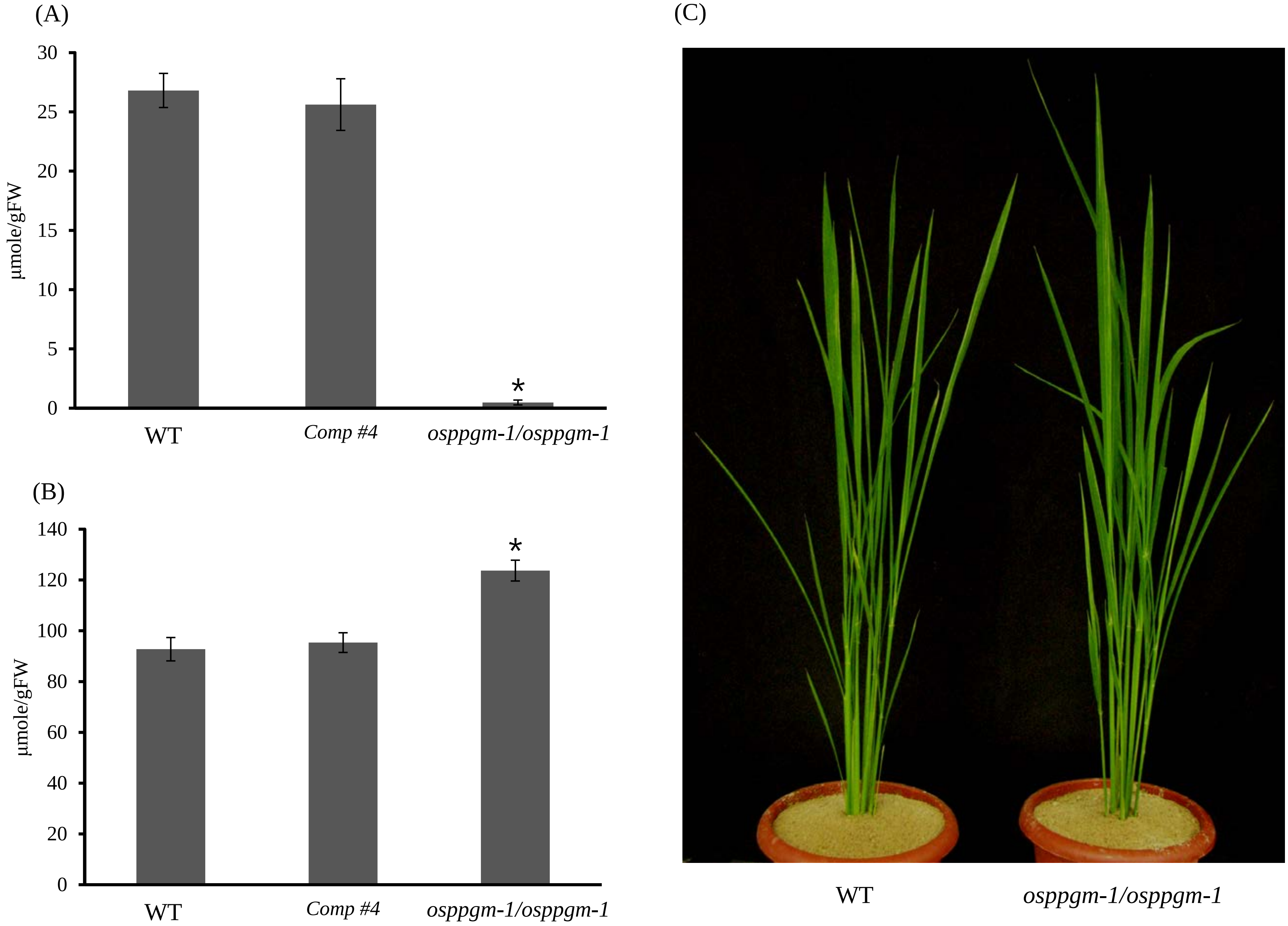

Fig. S2. Metabolite and phenotypic analysis of *osppgm-1/osppgm-1* mutant rice plant generated by anther culture.
(A, B) Starch (A) and sucrose (B) contents in *osppgm-1/osppgm-1*, wild type and Comp #4 plants grown for 12 weeks. Each data point represents the mean (±SD) from at least three different plants. *, $P < 0.01$. Second leaves from top of the plants harvested at the end of the day were analyzed. (C) Growth phenotype of eight-week-old plants grown in greenhouse conditions.

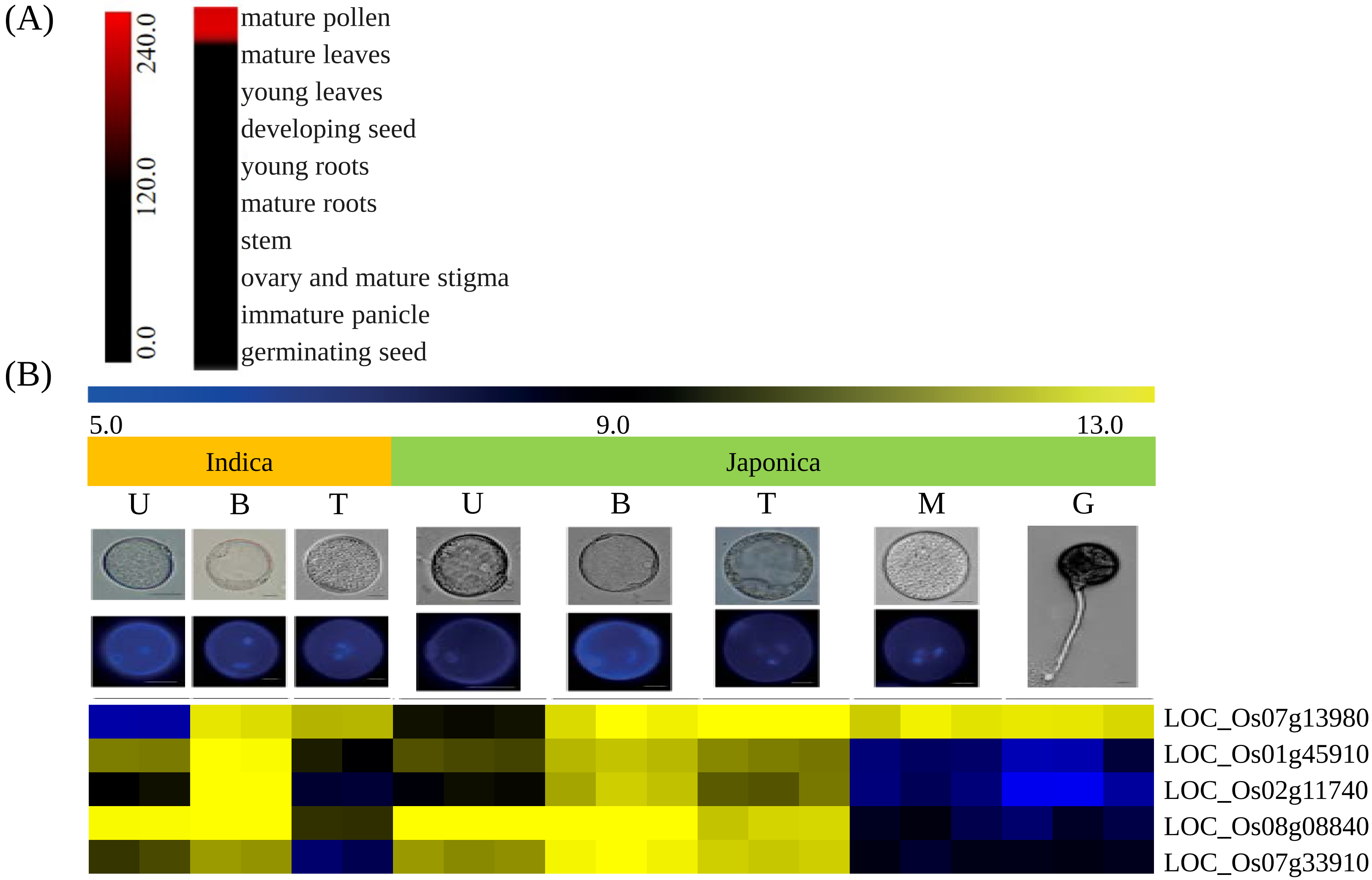

Fig. S3. Digital expression profile of *OsAGPL4*, the putative plastidic ATP/ADP translocator, and Glc-6-P/phosphate translocator genes. (A) Heat map generated with sum of abundance of *OsAGPL4* signatures (GATCAGAATCAACAAAG, GATCGAAATAAATACTG, and GATCTGAGTTCGACCTT) from the Rice MPSS database. (B) Heat map expression analysis of *OsAGPL4* (*LOC_Os07g13980*), plastidic ATP/ADP translocator (*LOC_Os01g45910* and *LOC_Os02g11740*), and Glc-6-P/phosphate translocator (*LOC_Os08g08840* and *LOC_Os07g33910*) genes using Affymetrix rice microarray data prepared from developing pollens of indica and japonica varieties downloaded from NCBI GEO (Nguyen *et al.* 2016. Genome-wide identification and analysis of rice genes preferentially expressed in pollen at an early developmental stage. Plant Molecular Biology, doi:10.1007/s11103-016-0496-1). B, bicellular pollen; G, germinating pollen; M, mature pollen; T, tricellular pollen, U, unicellular pollen stage. Note: Of four Glc-6-P/phosphate translocators previously predicted by Toyota *et al.* (2006), *LOC_Os07g34010* and *LOC_Os07g33960* are no loner present in the most recent Rice Genome Annotation Project Release 7 data.

Table S1. List of PCR primers used in this study

Names	Primer sequence
OspPGM-F	CTGCTCAGATTATCACTAAAATTG
OspPGM-R	AGAGCTAGGTCTATTAAAGGCTTC
OscPGM-F	ACGATATGACTATGAGAATGTTGA
OscPGM-R	AGATGTAAAAAGGCTATCAAACAG
OsAGPL1-F	TTCCTTTTCTTGATCTCTCTTCTA
OsAGPL1-R	CTCCTGACAAGATTAAAATGTGTT
OsAGPL2-F	AGAGGTTGATGGAAAGATTGAATA
OsAGPL2-R	CTGATCTCCACACAAGATTACAAC
OsAGPL3-F	GCTAATAAACCAATGTACACATCA
OsAGPL3-R	GATGACTAATCCATCTGCAATTAT
OsAGPL4-F	GATATGGGAACTTTTGGTTTG
OsAGPL4-R	TGCAGTTTTCTACTTTCGTTG
UBQ5-F	GACTACAACATCCAGAAGGAGTC
UBQ5-R	TCATCTAATAACCAGTTCGATTTC
PF1	CTATGTAGAATAGCTGTTTTGCACCCAAC
PR2	TATAGCACTACGGGCATATTCTTCATTTG
PF2	TGCTGCTAGGAACCAAGAGTAAATCAATA
PR2	TCAAACACAAGATGAAATGGGAAATAAAC
LF1	TGCATATGTTAAATCATCCACGGTTTTAT
LR1	ACCAATAACAGAGCGATCAACACTACATT
G1	ATCCAGACTGAATGCCCACA
L1	CGATTTTTGAAATGCGAGAGCG
